# Health Tract, No. 63

**Published:** 1861-12

**Authors:** 


### HEALTH TRACT, No. 63.

---

### SKATING

Is one of the most exhilarating of all pastimes, whether on the ice, or over our parlor or hall floors, with roller-skates. In the days of "Queen Bess," some three hundred years ago, it was a favorite amusement with the Londoners, whose facilities for the same were limited to pieces of bone attached to the shoes. As lives have been lost in connection with skating, the following suggestions are made :

1. Avoid skates which are strapped on the feet, as they prevent the circulation, and the foot becomes frozen before the skater is aware of it, because the tight strapping benumbs the foot and deprives it of feeling. A young lady at Boston lost a foot in this way; another in New-York, her life, by endeavoring to thaw her feet in warm water, after taking off her skates. The safest kind are those which receive the fore-part of the foot in a kind of toe, and stout leather around the heel, buckling in front of the ankle only, thus keeping the heel in place without spikes or screws, and aiding greatly in supporting the ankle.

2. It is not the object so much to skate fast, as to skate gracefully ; and this is sooner and more easily learned by skating with deliberation ; while it prevents overheating, and diminishes the chances of taking cold by cooling off too soon afterward.

3. If the wind is blowing, a vail should be worn over the face, at least of ladies and children ; otherwise, fatal inflammation of the lungs, "pneumonia," may take place.

4. Do not sit down to rest a single half-minute ; nor stand still, if there is any wind; nor stop a moment after the skates are taken off; but walk about, so as to restore the circulation about the feet and toes, and to prevent being chilled.

5. It is safer to walk home than to ride ; the latter is almost certain to give a cold.

6. Never carry any thing in the mouth while skating, nor any hard substance in the hand ; nor throw any thing on the ice ; none but a careless, reckless ignoramus, would thus endanger a *fellow*-skater a *fall.*

7. If the thermometer is below thirty, and the wind is blowing, no lady or child should be skating.

8. Always keep your eyes about you, looking ahead and upward, not on the ice, that you may not run against some lady, child, or learner.

9. Arrange to have an extra garment, thick and heavy, to throw over your shoulders, the moment you cease skating, and then walk home, or at least half a mile, with your mouth closed, so that the lungs may not be quickly chilled, by the cold air dashing upon them, through the open mouth ; if it passes through the nose and head, it is warmed before it gets to the lungs.

10. It would be a safe rule for no child or lady to be on skates longer than an hour at a time.

11. The grace, exercise, and healthfulness of skating on the ice, can be had, without any of its dangers, by the use of skates with rollers attached, on common floors ; better if covered with oil-cloth. Lessons are given in this pleasant and exhilarating exercise at Mr. Disbrow's on Fifth Avenue, whose spacious and well-conducted establishment ought to be well patronized.

